# Nature’s “Free Lunch”: The Contribution of Edible Insects to Food and Nutrition Security in the Central Highlands of Madagascar

**DOI:** 10.3390/foods10122978

**Published:** 2021-12-03

**Authors:** Jochen Dürr, Christian Ratompoarison

**Affiliations:** 1 Center for Development Research (ZEF), University of Bonn, 53113 Bonn, Germany; 2 Department of Food Sciences and Technology, Campus of Ambohitsaina, College of Agricultural Sciences, University of Antananarivo, P.O. Box 175, Antananarivo 101, Madagascar; christianratompoarison@gmail.com

**Keywords:** entomophagy, insect consumption, protein intake, rural areas, Sandrandahy

## Abstract

Edible insects are a healthy, sustainable, and environmentally friendly protein alternative. Thanks to their quantitative and qualitative protein composition, they can contribute to food security, especially in Africa, where insects have been consumed for centuries. Most insects are still harvested in the wild and used for household consumption. So far, however, little attention has been paid to insects’ real contribution to food security in low-income countries. Entomophagy, the human consumption of insects, is widespread in many rural areas of Madagascar, a country, at the same time, severely affected by chronic malnutrition. This case study was carried out in a region where entomophagy based on wild harvesting is a common practice and malnutrition is pervasive. The data were obtained in 2020 from a survey among 216 households in the rural commune of Sandrandahy in the central highlands of Madagascar. Descriptive statistics, correlation, and regression analysis were used to show the relative importance of insects for the local diet and to test various hypotheses related to food security. Results show that insects contribute significantly to animal protein consumption, especially in the humid season, when other protein sources are scarce. They are a cheap protein source, as much esteemed as meat by the rural population. There are no significant differences in the quantities of insects consumed by poorer versus richer households, nor between rural and urban households. Insect consumption amounts are strongly related to the time spent on wild harvesting. The importance of edible insects for poor, food-insecure rural areas and how entomophagy can be promoted for better food and nutrition security are discussed.

## 1. Introduction

Already, in the 1970s, insects were proposed by Meyer-Rochow as a solution to the world protein shortage [1]. At the same time, Chavunduka [2] claimed that insects are a cheap source of protein in Africa. In the early 21st century, there has been a renewed interest in edible insects’ possible contribution to food and nutrition security [3], especially as an important protein source [4]. Worldwide, insects are mostly harvested in the wild and are predominantly consumed within the household. Depending on the local context and traditions, insects play a significant role for local diets, especially in times of food shortages [3,5,6]. Edible insects are healthy, sustainable, and environmentally friendly protein alternatives [7]. Thanks to their quantitative and qualitative protein composition, they can contribute significantly to food security, especially in Africa, where insects have been consumed for centuries [8,9], with exceptions such as Ethiopia, where insects appear much less appreciated than elsewhere in Africa [10]. Proteins are the main components of an insect’s body, representing 35% to 77% of its dry matter [11]. In addition, the digestibility of insect proteins is very high for many preparations. As an indication, for the house cricket, which is popular for rearing (but not very popular as a food item in Madagascar [12]), the digestibility is between 78% and 83% [13]. Some insect species contain considerable amounts of essential minerals (K, Na, Ca, Cu, Fe, Zn, Mn, and P), as well as vitamins from group B and vitamins A, C, D, E, and K [14,15,16]. Moreover, insects contain important mono- or polyunsaturated fatty acids recommended by nutritionists. However, when considering entomophagy for nutrition security, one also has to bear in mind the possible contamination of edible insects by pathogens or pesticides, and the presence of allergic proteins and anti-nutrients [17].

Nevertheless, the importance of insects for food and nutrition security cannot be estimated yet on a global scale, and there are only a few specific studies available for the local level [3]. Moreover, insects are not only consumed in the case of food scarcity, but also because, and perhaps more importantly, they are considered a traditional and delicious food item [18].

Madagascar still faces severe problems of chronic malnutrition and stunting. Average protein supply is only 43 g/capita/day [19], one of the lowest per capita supplies worldwide, and not enough to ensure the recommended daily intake (of 0.75 g proteins per kg body weight of adults, see [20]) for all Malagasy. Insufficient intake of animal proteins is a major nutritional problem in the country [21]. Moreover, micronutrients, such as iron, are not sufficiently consumed; around one third of women and men in Madagascar are anaemic [22]. At the same time, entomophagy is still a widespread phenomenon in many parts of Madagascar. Insects are a commonly used food of the rural population. Nevertheless, there are no studies showing the actual amounts of consumed insects [12]. As there are no public statistics of insect consumption in terms of quantities or nutritional values, or of the socioeconomic background of insect consumers, it is hardly possible to know the real contribution of insects to food security in Madagascar. Besides, there are concerns that the potential of entomophagy is in danger because of decreasing insect supply, possibly because of habitat loss due to slash-and-burn practices and forest overuse [23], but there are also no data available to confirm this declining trend.

Some studies have focused on identifying the insect species consumed by local communities, using survey methods with questionnaires or semi-structured interviews and taxonomy. For example, Agbydye et al. [24] found that termites, the large African cricket, and the pallid emperor moth were the most frequently consumed insect species in Benue state of Nigeria. Bomolo et al. [25] found 11 species consumed in Haut-Katanga Province of Congo, and different proportions of groups eating these species. Caterpillars and termites were among the most consumed insects, but the results depended significantly on the age, ethnicity, family status, and education level of the respondents. These studies showed the wide variety of insect species consumed, but did not quantify the respective amounts.

So far, edible insects have not been included in national consumption surveys [26], and only one nationally representative survey was carried out accounting for insect consumption. This survey [27] showed that, in Laos, the most frequently consumed insects are weaver ant eggs, crickets, grasshoppers, cicadas, and bamboo worms. Almost 97% of people consume insects, and 44% of the population does this (very) frequently. However, the survey did not quantify the consumption amounts and, hence, cannot specify the nutritional intake of insects and their importance to food and nutrition security.

There are some studies on the regional or local level that state the amounts of insects consumed. Using 24-h recall, Yhoung-Aree et al. [28] found that between 2 and 32% of schoolchildren in Pana District, Northeastern Thailand, consumed, depending on the season, between 2 g and 26 g of insects per person per day, mainly silkworm pupae, crickets, and beetles. Acuña et al. [29] reported approximate monthly amounts of edible insects consumed as part of the traditional food system of a Popoloca village in Puebla State, Mexico. The quantities vary according to weather conditions, individual preferences, and the prevalence of specific species and are, therefore, difficult to estimate. Between February and September, a whole family gathers around one to two litres of different species from different orders, once or twice per season, and individual persons collect around 12–15 larvae, also once or twice per season. However, the nutrient intake, especially the protein intake, resulting from the amounts consumed were not calculated by these studies. One exception is the study of N’Gasse et al. [30], who report that, in the district of Bangui in the Central African Republic, 29% of the annual animal protein intake comes from the consumption of caterpillars.

Given the scarce evidence on the importance of insects as a protein source, our central objective was to use a case study in the central highlands of Madagascar to analyse how much entomophagy actually contributes to local food and nutrition security. The originality of this case study lies, on the one hand, in its comprehensive empirical assessment of the above-mentioned food security topics: firstly, by showing how much insects are contributing to local diets as a protein source, and how this varies seasonally; secondly, by examining the relative importance of the insect protein source in relation to other animal and plant protein sources; and, thirdly, by evaluating if insects are a cheaper protein source compared to other protein sources. On the other hand, we tested three hypotheses related to important food security issues: 1. Poorer households consume more insect protein compared to richer households. 2. Significant differences in protein consumption patterns exist between villages, and between rural and urban sites. 3. Insect protein consumption is positively related to farm size and harvesting time.

Better knowledge about the contribution of insects to food and nutrition security could foster sustainable ways of promoting entomophagy in different parts of Madagascar. Even if between regions, and even within regions, different sorts of edible insects are consumed in different numbers [23], and a generalisation of specific case study results is therefore difficult, there are some general conclusions this paper will draw, which can be tested in further studies: firstly, the importance of edible insects as a protein source in poor, rural areas where entomophagy is common; and, secondly, the fact that, as long as entomophagy is based on wild harvesting, its contribution to food and nutrition security remains restricted by seasonality and natural conditions, and by the time that can be spent on the collection of insects.

The rest of the article is organised as follows. In chapter two, we describe the study site and explain our data sampling process. Chapter three presents the descriptive results concerning consumption amounts of insects, protein intake, and the cost of insect proteins compared to other animal and plant proteins. In chapter four, the hypotheses are tested. Then, we interpret the results in the discussion section (chapter five). Conclusions for the promotion of insect consumption for better nutrition are finally drawn (chapter six).

## 2. Materials and Methods

### 2.1. Research Site

The research was carried out in the southern zone of the central highlands of Madagascar, in the Amoron’i Mania region, district of Fandriana, rural commune of Sandrandahy (20°21′00″ S, 47°17′42″ E, see the map in the Appendix A), where the ProciNut project “Production and Processing of Edible Insects for Improved Nutrition” is being undertaken. Sandrandahy consists of 38 fokontany (villages), with a total of 5055 households. The altitudes vary between 1200 and 1500 m.a.s.l., and the tropical climate has two well-marked seasons—a hot and humid one, from October to April, and a cool, dry one, from May to September [31]. Agriculture is the chief economic activity there, rice being the main staple crop. Poverty is widespread and per capita income in 2010 was only Madagascar Ariary (MGA) 672 thousand (at the time, around Euro (EUR) 272) per annum (or EUR 0.65 per day; [32]). Stunting and chronic malnutrition is pervasive, reaching 64% and 69%, respectively, of the population [33]. The widespread and chronic food and nutritional insecurity is aggravated after October, and is especially strong in the months of January and February, when rice reserves are dwindling. The main dish consists of rice, complemented by beans, vegetables, and sometimes meat or fish. According to FAO [33], the underlying problems are the small landholdings coupled with demographic pressure on land, low number of livestock, low prices of agricultural produce, and an increase in food prices during the lean season, when the availability of rice is low. Given the characteristics of the commune, it represents an interesting case for studying the contribution of entomophagy to food security in a context of malnutrition and protein scarcity, a situation which is common in most parts of Madagascar.

### 2.2. Sampling Design

We used cluster sampling as a common probability sampling procedure. Each fokontany was considered as a cluster. As we knew the number of households of each of the 38 clusters, systematic sampling using probability proportional to size was possible, i.e., larger clusters have a proportionally higher likelihood of being selected than smaller ones. In order to keep the design effect at low levels, but also taking into account the difficulties of reaching the villages, we decided to take interviews in 12 of the 38 villages, so that the relation of number of villages (clusters) to number of households was in the range considered as “relatively safe” (maximum 40–50 households per cluster; see Magnani 1999, p. 18). We used the procedure described in Magnani [34] (p. 24) to select the 12 fokontany.

In January 2020, we conducted 18 interviews in each of the 12 villages—216 interviews in total. The measurement entity was the household, which was our unit of interest. For each of the 12 randomly chosen clusters, we were able to access a list of all households of the village. First, we randomly and systematically drew 18 households from the list. This was achieved by dividing the total number of households by 18 (planned number of interviews considered manageable given the available time and number of enumerators). The result was the sampling interval (SI). We started randomly at the top of the list at one of the households, number 1 to number (SI), and then chose every (SI)th household. We applied the same procedure in a second step to draw 12 additional households in the event that some of the 18 original households were absent or would decline to co-operate (which has happened in only two cases).

Data collection was carried out by face-to-face interviews at households with a standardised questionnaire. Preferentially, the head of each household and/or his/her spouse was interviewed to ensure that the respondents were aware of the consumption of the whole household. The sample comprised 66% of male and 34% of female respondents, 72% of whom were heads of household. Six local enumerators were recruited and trained on the questions before conducting the survey. The questionnaire was pretested to ensure a general understanding and a uniform questioning technique. The survey was announced beforehand by the village chiefs to instil confidence. Participation was voluntary and took place without remuneration. The survey software KoBoToolbox from the Harvard Humanitarian Initiative was used to collect the data. The answers were entered into a data acquisition app via smartphones, stored in a cloud, and then transmitted to computers. The questionnaire was initially developed in English, and then translated into Malagasy by the local project partners. The analyses were carried out with SPSS (version 27.0, IBM, Böblingen, Germany) and Microsoft Excel.

The main focus of the questionnaire was on quantitative data on the agro-socioeconomic context, insect harvest and consumption, and nutrition. In addition, qualitative data were collected to better understand local entomophagy practices. Qualitative questions included dichotomous questions (“Would you like to harvest more insects?”), as well as open-ended questions (“Why would you like to harvest more insects?”). The questionnaire was divided into five main parts: 1. General information; 2. Socio-economic status and agriculture; 3. Insect harvesting; 4. Insect consumption; 5. Food frequencies.

The survey was conducted with the approval of the Head of Amoron’i Mania Region, the prefecture of Ambositra District, the Mayor of Sandrandahy Commune, and the Chiefs of each fokontany. We informed the respondents that the data collected remain confidential, that they have the right to stop the interview at any point in time, and that all gathered information will be used only for research purposes. Respondents gave their oral consent to the data collection.

### 2.3. Seasonal Consumption and Harvesting Amounts

For the analysis of the contribution of insects to food and nutrition security, we used food frequency tables to gather consumption data of insects, as well as of other food items, for a whole year. First, it was examined in which months of the year (from January to December) every item is consumed. Then, it was asked how often and how much of the food is consumed each time, i.e., the quantities per day, week, or month, so that average monthly amounts could be calculated. Multiplying the monthly amounts by the number of months the food item is consumed gives the total annual amount. This procedure also allowed us to assess seasonal patterns of consumption. (Note that the monthly consumption amounts per individual household are the same for all months when the item is consumed by this household (zero otherwise); collecting monthly consumption data was considered too difficult. The seasonal consumption pattern is the result of adding the amounts of all households.) However, in the case of insects, the seasonality pattern relies on harvesting amounts, not on consumption amounts. (Again, monthly data on consumption were considered difficult to collect but, as there is hardly any buying or selling of insects, monthly harvesting amounts nearly equal consumption amounts.)

The amounts consider the consumption of the whole household; despite possible individual differences, when calculating per capita consumption, we simply divided the household amount by the number of household members. Similar to consumption amounts, harvesting amounts were calculated by the number of months during the year when insects are collected, multiplied by the average monthly harvesting amounts. For transformation into kg, we used a standardised transformation factor for each local unit (e.g., one cup of insects = 200 g).

In different parts of the questionnaire, we independently collected data on the harvesting, buying, and selling of insects. Considering that total consumption amount = amount harvested + purchased − sold, it was possible to cross-check the consumption with the harvesting data (excluding the possibility of transferring insects between households as gifts). Only in 5% of all cases is the difference between the two higher than the average consumption amount. For all insect groups except one, average consumption amounts differ less than one kilogram from harvesting amounts, and the total average harvesting amount is 10% (0.9 kg per household) higher than the consumption amount, which results in rather conservative estimations about the consumption of edible insects.

### 2.4. Nutritional Composition and Costs of Insects and Other Foods

In the survey, people used local terms for insects, which normally do not denominate specific species, but often a certain stage in the life cycle of a certain insect family or order. For example, *Sakivy* denominates larvae, and *Abado* pupae of Coleoptera. The insect species, families, and orders belonging to each local name were identified by an entomologist of the ProciNut project. To determine the nutritional composition of the groups of species identified, bibliographic research was carried out. The keywords used were the scientific names of the species. The nutritional composition used for the locally denominated insects is given in Table A1 in the Appendix A. In some cases, an average protein content was calculated when data were available from different sources. In two cases (snout beetles and wild silkworm), where no data could be found, nutritional composition was used from different families belonging to the same order. In cases where nutritional information was only available for dry matter (beetle larvae and cicadas), an average moisture content of 70% was applied to calculate the protein content for fresh matter. In order to compare the protein content of commonly consumed animal- and plant-based food items, we used the tables given by the National Bureau of Nutrition (ONN) of Madagascar (see Table A2 in the Appendix A).

The protein costs were calculated by the average prices paid by the respondents per kg of purchased food item, and its protein content, described above. As most insects are not bought, we only obtained sufficient price data for two types of insects (adult scarab beetles and silkworms).

## 3. Descriptive Results

### 3.1. Socio-Demographic Characteristics

The average size of the interviewed households is 5.5 members. Nearly half of household members (49%) belong to the working age population, defined as 16–64 years old, meaning that the dependency ratio is around one. Ethnically, the population is quite homogenous: 95% of the interviewees are members of the Betsileo group; the rest belong to the Merina group, except for one respondent from Sakalava group. Most of the communities’ members (75%) have lived already for a long time in their villages; 25% of households have moved to their community only after the year 2000. With regard to the level of education of the interviewees, it can be stated that 4% were not enrolled in school, 56% have completed primary education, 25% have passed college, 12% achieved high school level, and 3% went beyond this level. Regarding religious beliefs, 61% stated to be Catholic, 26% Protestant, 12% Lutheran, and 2% attend the free churches. In total, 10% of the sample are female-headed households. However, all of these socio-demographic variables are not significantly correlated with insect protein consumption (household size, dependency ratio) or the mean values of insect protein consumption per capita of the different groups (long vs. recently established, female vs. male-headed, Betsileo vs. non-Betsileo, education levels, and religious beliefs) are not statistically different.

### 3.2. Agriculture and Income

Subsistence agriculture is predominant in Sandrandahy. According to our survey, farm households own, on average, 1.8 ha of land, of which 0.5 ha is irrigated for rice production. Paddy is the main crop in the region; on average, farmers produce 836 kg per year. One quarter of households sell rice, while the large majority (76%) only use it for home consumption. Rice, in terms of production value, accounts for 50%, followed by cassava (19%), sweet potato (9%), and corn (5%). In other words, more than 80% of production value comes from staple crops, whereas legumes, such as groundnuts, Bambara nuts (*Vigna subterranea*), and beans, together cover around 10%, and fruit and vegetables make up 4%. Most households own livestock: on average, they have two zebus and one pig, around 15 chicken, and three ducks. Moreover, 58% of households catch fish in their rice fields.

Households complement their agricultural activities with non-farm activities to make ends meet. Actually, 96% of households interviewed earn some sort of non-farm income, mainly from low remunerated casual work. Non-farm activities account for 53% of cash income, whereas livestock selling contributes 39% and crops around 7% of total cash income. On average, cash income is around EUR 120 per capita per year (EUR 0.32 per day), of which only around EUR 20 is spent on food.

### 3.3. Harvesting and Consumption of Edible Insects

In the rural commune of Sandrandahy, wild harvesting for home consumption dominates. Insects are rarely bought (2% of total consumption amount) or sold (0.2%), so that consumption nearly exclusively depends on the collection of insects. The preferred insects are adult beetles from the family of Scarabaeidae (*Voangory*), harvested and eaten by 87% of all households, followed by cicada (30%), pupae of Coleoptera (*Abado*, 29%), and locusts (21%) (see Table 1). The *Voangory* are adult scarab beetles that stay in the ground during the day, from which they crawl out and fly away after sunset. They are then found in larger clusters near rice fields or in the grass or shrubs where they can be easily caught. Larvae and pupae of silkworm are not much consumed (3%), as no tapia trees, the natural host plant of the wild silkworm (*Borocera cajani*), are found nearby, and domesticated silkworm (*Bombyx mori*) production is not important in the region. Locusts and crickets are collected on a rather irregular basis, for example, when there is an infestation. In quantitative terms, adult scarab beetles dominate consumption, as 66% of the total amount of insects consumed (as measured in kg) come from the *Voangory* type. The average and total amounts of other species are comparatively low, because only a few households consume them. However, those households which consume these species can consume relatively large amounts. Almost all (95%) of the households consume insects. The average consumption is 9.0 kg per household per year, which corresponds to 1.7 kg per capita, but varies greatly (Standard Deviation (SD): 10.6/2.0 kg).

In general, insects are consumed for two main reasons: people find them tasty (88% stated that they have a good taste) and it is a traditional food (65%, multiple answers possible). Moreover, insects are considered by the local population as a full meat substitute: they are predominantly consumed as a main dish with rice (92% of households harvest insects for this reason), and only sometimes as snacks (7%). Moreover, when asked whether they would choose a plate of insects over a plate of meat, if offered both for free, 52% responded that they would choose the latter, 37% would prefer insects over meat, and 11% like meat and insects equally. Around three quarters of households would like to consume more insects and are, therefore, interested in starting to rear them.

In total, 82% of households would like to collect more insects. The reasons why they do not harvest more refer much more to a lack of insects (“they are rare”, 52%; “rare because of climate and habitat change”, 27% of answers) than to labour constraints (“too busy to catch more”, 12%). Moreover, 81% of interviewees reported a diminishing trend of harvested insect quantities in recent years.

### 3.4. Seasonality of Harvesting and Consumption

**Figure 1:**
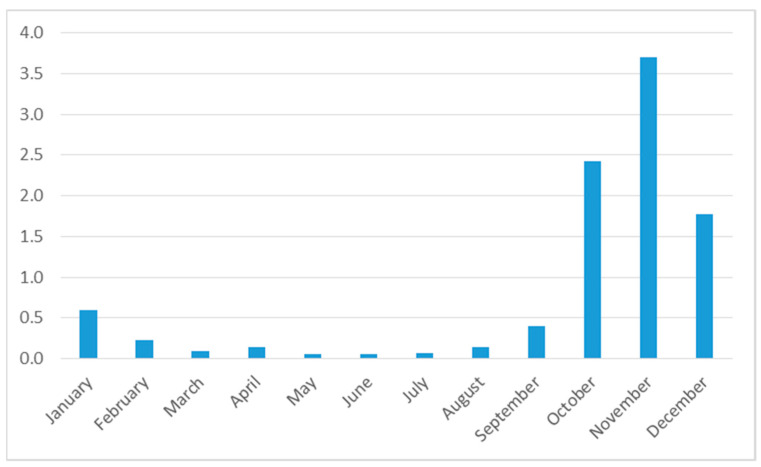
Monthly harvesting amounts of edible insects, in kg per household. Source: own calculations based on survey data.

Many edible insect species are only seasonally available [18]. However, different species appear at different times of the year, and there are some insects which are available year-round. It is not clear how seasonal availability translates into seasonal patterns of consumption. As Figure 1 shows, however, there is a clear pattern in Sandrandahy: the harvesting of insects is highest between October and December. (As explained above, we did not collect data on monthly consumption of insects but, as the households consume nearly all insects immediately and hardly any insects are bought, sold, or preserved, seasonal consumption should follow seasonal harvesting patterns.) Some 82% of the total amount is collected in these three months, which are, at the same time, the start of the lean season. This is explained by the fact that the most commonly consumed species—adult scarab beetles, but also cicada—only appear in these months, whereas other species that are available year-round, such as locusts, crickets, and the larvae of Coleoptera, are not collected much.

**Figure 2:**
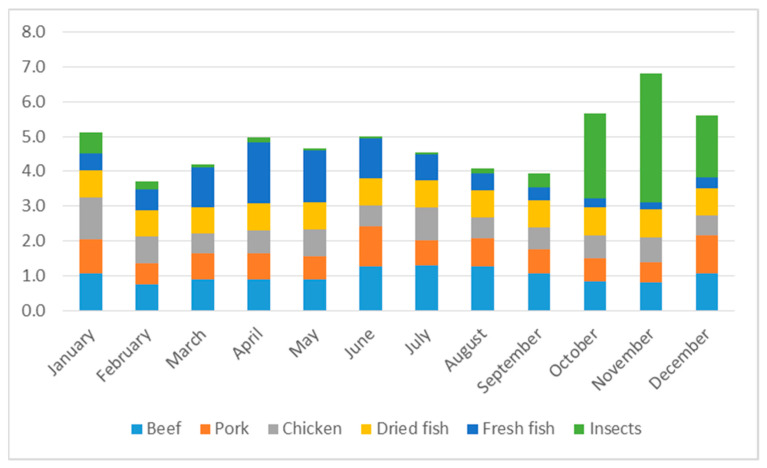
Monthly amounts of meat and fish consumed and edible insects harvested, in kg per household. Source: own calculations based on survey data.3.5. Protein Intake.

For comparison, Figure 2 shows the monthly amounts (in kg) of beef, pork, chicken, and fish consumed by households over the year, as well as insects. The consumption of meat fluctuates only slightly throughout the year (Coefficient of Variation (CV) of 15%), whereas that of (fresh and dried) fish varies more (CV of 32%), both together reaching their highest levels between April and June, diminishing afterwards to reach their lowest levels in October and November, exactly the months when insect harvesting is at its peak. Between October and December, insects constitute 44% of the total amount of meat, fish, and insects consumed, meaning that edible insects (over-)compensate for the lower levels of meat and fish consumption. Furthermore, due to insect consumption, this is the period with the highest total consumption of meat, fish, and insects.

### 3.5. Protein Intake

**Figure 3:**
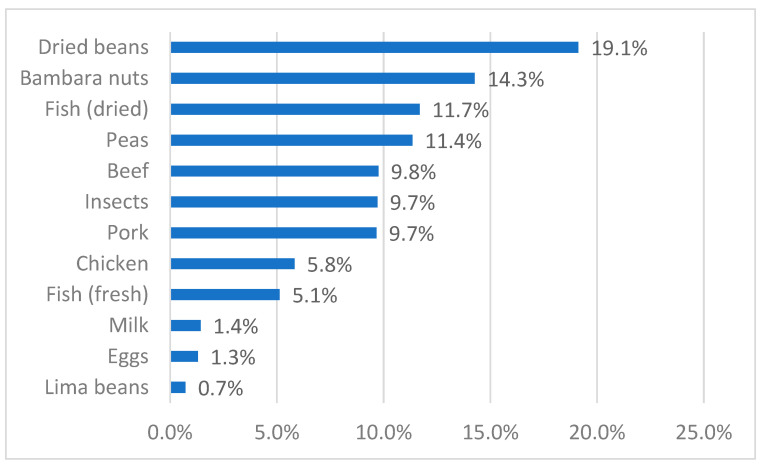
Average protein intake of different sources, in % of total. Source: own calculations based on survey data and data from Table A1 and Table A2 in the Appendix A.

The quantities of meat, fish, and insects shown in Figure 2, and of protein-rich crops were converted into amounts of protein consumed by taking the average protein content of the different sources (see Table A1 and Table A2 in the Appendix A). In general, animal protein sources are limited: an average household of 5.5 members ingests 69 kg of animal products (including insects) per year, containing 9.9 kg of proteins (SD 6.2 kg), of which 1.8 kg comes from insects (SD 2.0 kg). This equals an average per capita animal protein consumption of around 5 g/day (not differentiating between adults and children, male and female), lower than the average national supply of 9 g/day [19]. Besides animal products, protein-rich crops, mainly legumes (beans, bambara nuts, peas), are consumed. The protein coming from these plants is 8.3 kg per household, not much lower than that of animal products. Figure 3 shows the different sources of proteins in the region. Insects are responsible for nearly 10% of protein consumption of the rural population, in the same range as pork and beef.

However, probably the most important protein source is rice (not considering that rice is not the best protein source, as it contains less essential amino acids), which is eaten on a daily basis. To illustrate, paddy production for personal consumption, which is on average 737 kg per household (we only collected production data, but not consumption data of rice, corn, and other cereals), contains around 44 kg of protein per year per family, compared to just 18 kg coming from animal products and legumes. Per capita protein supply from animal products, legumes, and rice together is around 31 g/day, lower than the national average of 43 g [19].

### 3.6. Cost of Different Protein Sources

**Figure 4:**
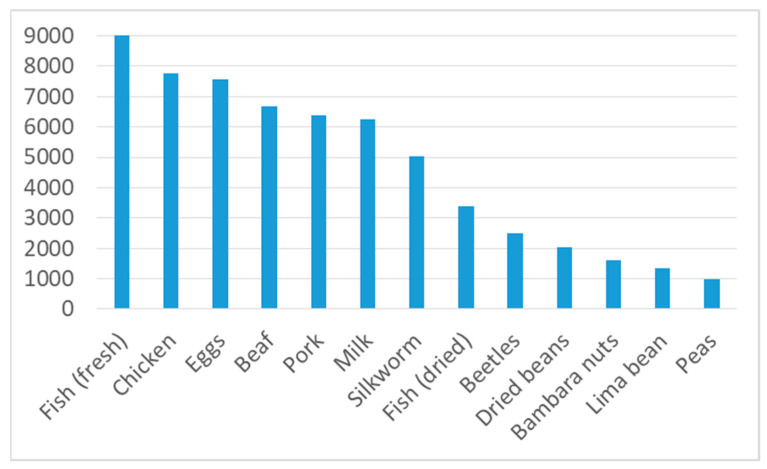
Protein cost of different foods, in MGA (at the time of the survey, EUR 1 = MGA 4000 (www.oanda.com), accessed on 5 May 2021) per 100 g of protein. Source: own calculations based on survey and data from Table A1 and Table A2 in the Appendix A.

It has been assumed that insects are a cheaper source of macro- and micronutrients than meat [3]. For our study region, Figure 4 demonstrates that meat, as well as milk and eggs, are indeed a relatively expensive source of protein: 100 g of their protein costs between MGA 6000 and 8000 (EUR 1.50 to 2.00). Fresh fish is the most expensive source (around MGA 9000 or EUR 2.25 per 100 g), whereas dried fish protein is much cheaper (nearly MGA 3400 or EUR 0.84 per 100 g). The calculated protein price of insects varies according to the species (as mentioned above, we only have sufficient price data for two types of insects). Whereas protein from adult scarab beetles is relatively cheap (around MGA 2500 or EUR 0.63 per 100 g of protein), that from silkworm chrysalis is at MGA 5000 (EUR 1.25), twice as expensive. In general, plant-based proteins are much cheaper (around MGA 1000 to 2000 or EUR 0.50 to 1.00/100 g) compared to animal protein sources.

As almost all insects are harvested in the wild by family members, there are hardly any cash expenses involved. The costs inferred are only the opportunity costs of harvesting them. The mean time to collect one kilogram of insects is, according to our data, 6.6 h; if this time could be used for other productive purposes instead, this would be an opportunity cost. This might differ from household to household but, assuming an opportunity cost that equals the agricultural minimum wage of MGA 675/h (https://www.minimum-wage.org/international/madagascar, accessed on 3 June 2021), collecting one kilogram of insects would have an opportunity cost of around MGA 4500, or approximately MGA 2250 (EUR 0.56) per 100 g of protein (considering a weighted average of 20 g protein/100 g of fresh insect weight), a bit lower than the price of protein from adult beetles calculated above. However, as more than two thirds (68%) of the harvesting is done by children and youths (5–15 years old), the opportunity costs are probably lower (assuming that insect collection is not done at the expense of school attendance).

### 3.7. Micronutrient Intake

Many authors have been highlighting the contribution of entomophagy to the protein intake in protein-scarce food environments. However, edible insects’ contribution to micronutrient intake might be even more important in some cases [35]. Especially in low-income countries, many people, especially children and women of reproductive age, suffer from iron and zinc deficiencies, which represent two of the most severe micronutrient deficiencies globally [36]. Adult scarab beetles (*Holotrichia* sp.), the far most consumed insect in our project region (*Voangory*), have an iron and zinc content of 9.1 mg and 8.8 mg/100 g edible portion, respectively (Köhler et al. 2019). As the daily required intake of children, women of reproductive age, and men is 10 mg, 18 mg, and 8 mg/day for iron, and 5 mg, 11 mg, and 8 mg/day for zinc [37], respectively, a family consisting of one couple with three children would need 34 mg of zinc and 56 mg of iron per day. The actual intake of 5.92 kg beetles per family would only allow for around 10 (iron) to 15 (zinc) days of the total required intake. As these beetles are consumed only seasonally (1–3 months), at least over that time, they can contribute substantially to the daily required intake of these important micronutrients

## 4. Hypotheses

### 4.1. Poorer Households Consume Significantly More Insect Proteins Compared to Richer Households

Given that insects are a cheaper source of protein compared to other animals, to the extent that they are almost such a thing as a “free lunch” [38], it can be supposed that poorer people will rely more on this protein source than richer ones. We used cash income as a proxy for poverty. As few people buy insects, we do not expect any direct positive effect of income on insect protein consumption, but one on the consumption of other animal protein sources (meat, fish, milk, eggs), which could then indirectly lead to lower insect consumption of richer households, in relative and/or absolute terms. Table 2 shows the correlation between the variables: no relationship could be established between insect protein consumption (in kg per capita per year) and per capita cash income, but a relatively strong correlation (*r* = 0.546 **) between income and other animal protein consumption. This means that richer households eat more meat and fish, but not less insects compared to poorer households. Insect and other animal protein consumption are not significantly correlated. It seems that households do not ingest less insect proteins only because other animal protein sources are more available, or, in other words, people with less income and less meat and fish consumption do not compensate this by higher (absolute) insect consumption. However, as poorer households have less access to meat and fish protein, the share of insects in their protein consumption tends to be higher compared to higher cash income households (*r* = −0.177 **). Finally, the more proteins from meat and fish are consumed, the lower the share of insect protein (*r* = −0.288 **).

### 4.2. Significant Differences in Protein Consumption Patterns between Villages, and between Rural and Urban Sites Exist

Different market access for animal products and different occurrence of insects for wild harvesting might lead to locally diverse protein consumption patterns in the 12 villages of our sample. Still, we found few differences between the places concerning per capita insect protein consumption: ANOVA post hoc tests (alpha = 0.05) only showed two different groups of villages, seven out of the twelve villages being in both groups. Concerning animal protein consumption, only two homogeneous groups could be identified: the (semi-)urban fokontany of Sandrandahy, with a higher consumption level, and the other villages. We did not find other patterns of low versus high consumption: some villages have below total average animal protein consumption and below total average insect protein consumption (4 out of 12). There are others with lower animal, but higher insect protein consumption (3), there are those with higher animal, but lower insect protein consumption (4), and, finally, one village with higher than average consumption of both protein sources. Consequently, there is no significant correlation between average animal protein and average insect protein consumption per village (*r* = −0.287, *p* = 0.365).

### 4.3. Insect Protein Consumption Is Positively Related to Land Size and Harvesting Time

As insects are mainly collected in the wild, bigger households might be able to collect and consume more insects than smaller households. Table 3 shows that insect protein consumption per capita is significantly correlated with the amount of time spent on insect harvesting, but not with household size. However, household size and number of hours spent on harvesting are positively correlated. This means that larger households have more labour available to catch more insects. However, this does not translate into higher per capita consumption in larger households because of the counteracting effect of the greater number of consumers in these households.

Since insects are collected in the fields, larger farms should have more space to search for wild insects and, hence, could harvest and consume more. Table 3 shows that farm size is indeed positively correlated with insect protein consumption, and correlates significantly with time spent on harvesting. It seems that, if more land is available, this opportunity is used by spending more time on harvesting insects, which allows for higher consumption.

Finally, we tested all three hypotheses together using a double-logarithmic regression model, with the dependent variable being protein per capita consumption. One influential case (Cook’s d) was excluded. The model explains 54% of variances, which is a good fit (see Table 4).

Table 5 presents the coefficients of the logarithmic variables. Because of heteroscedasticity problems (Breusch-Pagan test statistics: chi-square = 210.7, *p* = 0.000) and non-normal distribution of residuals, we estimated the parameters using robust standard errors (HC3 method). Only two variables have significant *p*-values: animal protein consumption (*p* = 0.003) and harvesting time (*p* = 0.000), whereas cash income and household size, but also farm size (which has a weak but significant correlation coefficient, see Table 3), have nonsignificant *p*-values. The variable harvesting time shows the highest partial Eta-squared of 18, much higher than that of animal protein (0.04), meaning that the former variable has the highest effect size and explains most of the regression results. For example, if we run a regression only including harvesting time as an independent variable, R square is still as high as 506 (*F* = 218.015, *p* = 0.000), whereas, if we run the regression with all other variables, excluding harvesting time, R square is merely 0.073 (*F* = 3.281, *p* = 0.007). It seems that the most important determinant of insect consumption is the time spent on collecting them, confirming part of hypothesis three.

## 5. Discussion

In our research area, entomophagy is widespread, with 95% of households consuming insects. This high prevalence of entomophagy is common in many African countries. For example, Anankware et al. [39] stated that more than 80% of interviewees in Ghana consume edible insects as a protein source. However, as explained above, not much quantitative evidence on insects as a protein source exists. In the commune of Sandrandahy, insects contribute nearly 10% to protein intake derived from animals and legumes, comparable with the proportions of beef or pork. Entomophagy as an important element of protein intake has only been documented in a few other studies. For example, Roulon-Doko, 1998, cited in [8], documented that insects consumed by the indigenous Gbaya people in the Democratic Republic of the Congo contribute to 15% of total protein intake.

However, the amounts of insects consumed and, therefore, of proteins coming from insects vary widely between households in our study. One explanation could be that poorer households consume more insects, substituting meat or fish proteins. However, in contrast to meat and fish, of which poorer households consume less, insect protein consumption is not correlated with household income level. This finding is consistent with the results of Manditsera et al. [6], who did not find a significant association between income and insect consumption in rural areas of Zimbabwe, but contrasts with the outcome of the study by Kisaka [40] for rural Kenyan households, where increased income leads to higher consumption of winged termites. For urban households, Kisaka [40] reports lower termite consumption, possibly because of better access to other protein sources, such as meat. In our study, meat and fish consumption is not correlated with edible insect consumption, meaning that households that can afford to buy more meat and fish continue to consume insects. This may be because total protein intake is still low, even for richer households, which continue using insects as a complement to their diets. In addition, insects are considered a free and easily accessible food, which everyone still uses, independently of the income situation. Finally, it might just be because most people, richer as well as poorer people, find insects tasty. Hence, it seems that insects are not viewed as an inferior good in relation to meat and fish, and are not substituted when income increases, as repeatedly stressed by various authors and summarised in the review by Meyer-Rochow et al. [17].

Even if insects are cheaper than meat and fish, insects are hardly bought by the rural population, but almost exclusively harvested in the wild. Hence, households’ collection of wild insects seems to be key in explaining consumption patterns in rural areas. The time dedicated to harvesting is strongly correlated with amounts of insect proteins consumed and explains most of the regression results. This seems obvious in a situation where consumption depends almost entirely on wild harvesting. Especially in rural areas, access to insects in the wild greatly influences their availability for consumption [6]. However, the variable “harvesting time” was not considered in any other study we have found. On average, 34 h per month are dedicated to harvesting insects during season, but this varies widely (SD: 37 h). The question then might be: what influences harvesting time? Or, in other words, why do some households spend more time on harvesting than others?

We can only speculate on this: it might be (limited) labour availability, but also natural limitations. As seen above, most households would be willing to collect more insects if possible. The limitations mentioned refer more to lack of insects due to loss of habitat, especially in recent years, and less to labour constraints. Overexploitation of insects and changes in habitat (i.e., deforestation) have also been reported as a threat to entomophagy in other studies (summarised by van Huis [41]). Another explanation could be the interest and willingness of household members to collect insects. As children are the main collectors (68% of harvesting time), a fact which is confirmed by another study in Madagascar [12], it would be necessary to know what influences their harvesting activities. For example, school attendance might influence gathering time in communities where insects are mainly collected by children [42]. Moreover, besides the time spent, the amounts harvested per hour (i.e., the productivity) also differ greatly between households, a fact that might be explained by the diverse knowledge and skills (where to find and how to catch insects) of the gatherers, i.e., mainly of children.

Most insect collection is done seasonally, in up to three months of the year, and concentrated on adult scarab beetles (*Voangory*). On the one hand, this limits consumption opportunities. As different species normally appear at different times of the year, diversification could extend the availability of edible insects during the year. On the other hand, the main harvesting season for insects in Sandrandahy coincides with the period when other protein sources, such as fish, are rare, so that insects can compensate for this scarcity, contributing nearly half (44%) of meat, fish, and insect consumption in this period. A similar fact was found in the Lake Tumba region in Congo (at the time called Zaire), where Pagezy 1975, cited in [3], documented that, in some months of the year, when the occurrence of caterpillars is high, those of fish and game are low, and vice versa. High percentages (60%) of insects as an animal protein source during two months of the rainy season were also estimated by Paoletti et al. [43] for an Amerindian group of the Amazon. Muafor et al. [44] report that adult beetles consumed in Cameroon are only seasonally available, but constitute an alternative protein source to meat and fish in the months when they occur. This means that insect consumption can smoothen protein intake of rural households during the year. Other studies have also stressed the importance of some insect species, such as caterpillars, for seasonal food security [45].

Finally, some of the limitations of our study should be mentioned. Firstly, as a case study design with a strong regional focus was chosen, the results cannot be transferred to other regions of the country especially when considering that, in Madagascar, remarkable differences in insect consumption between different regions exist [23]. Second, an exact quantification of the consumption of edible insects is difficult due to the frequent recall bias [27], especially if the consumption quantities are recorded over a longer period of time. In addition, insects are mainly harvested by children, which makes it difficult for respondents to have an overview of how much is collected. As mentioned above (Section 2.3), we tried to check this by comparing the quantities harvested with the quantities consumed, both of which were asked independently in the questionnaire. Even if this comparison showed only minor deviations, we cannot rule out the possibility that both amounts are biased. Moreover, the frequencies of the other food items may have been affected by the recall bias. Third, we focused only on the protein content of insects (and some minerals found in *Voangory*), but did not consider, for example, fatty acids or vitamins. Moreover, we used data of protein content from the literature, which may differ for the insects found in the research area. Nutritional values depend not only on the species, but also on their diets and, hence, on their habitats, and on the processing methods [17]. Therefore, nutritional data on local insects and the influence of processing practices are required (and will be provided in the future by ProciNut project) in order to better assess their specific nutritional values. Fourth, the food safety aspect was not taken into account in our research. It is known that edible insects can be contaminated by micro-organisms, but also by pesticides. Even if contamination of microbes is reduced or eliminated by processing methods, such as boiling or deep-frying [17], further research is needed to ensure food safety of local insect consumption.

## 6. Conclusions

Apart from the general recognition of edible insects as an important protein source in developing countries [3], not much quantitative information on their contribution to food security is available. This study tried to partly fill this research gap by examining insect consumption in one rural commune in the highlands of Madagascar. Our results might be different in other rural areas. However, given the widespread tradition of entomophagy in this country [23], it can be concluded that entomophagy is a relevant and cheap local food source—a “free lunch”. This is especially the case in the lean season, when other protein sources are scarce. However, the seasonality of insect occurrence also limits the possible amounts for consumption purposes. Moreover, due to climate and habitat changes, insects have become rare. The decreased occurrence and limited availability of insects, and the fact that they are liked as much as meat by the local population, represents an incentive for starting to rear them. However, the type of insect that is most consumed in the region, the adult scarab beetle, is difficult to rear because of its long life cycle and underground life when in the larval stage. Commonly reared species, such as crickets, are not consumed much yet by the local population, but might be accepted by consumers when offered to them in other forms, for example, as cricket flour used in buns [46]. Given local demand and appropriate incentives, such as extension services and knowledge transfer, as well as start-up aid for investments, the raising of insects could become an interesting business for smallholders, as seen in other countries, such as Thailand [47]. This could allow food and nutrition security and the tradition of entomophagy in poor rural areas of Madagascar to be strengthened.

## Figures and Tables

**Table 1:** Annual consumption of insects by households (*n* = 216).

	Beetle (Larva) *Sakivy*	Beetle (Pupae) *Abado*	Beetle (Adult) *Voangory*	Beetle (Adult) *Voanosy*	Silkworm *Landibe Zana-dandy*	Locust *Valala*	Cricket *Akitra*	Cicada *Jorery*	Diving beetle *Tsikovoka*	Total
No. of consuming hh	8	62	187	27	6	45	2	65	4	205
% of all hh	4%	29%	87%	13%	3%	21%	1%	30%	2%	95%
Total amount in kg	34.2	114.2	1279.1	107.6	7.8	195.6	0.6	200.5	1.5	1941.0
% of total amount	2%	6%	66%	6%	0%	10%	0%	10%	0%	100%
Amount per hh in kg	0.2	0.5	5.9	0.5	0.0	0.9	0.0	0.9	0.0	9.0
Per consuming hh in kg	4.3	1.8	6.8	4.0	1.3	4.3	0.3	3.1	0.4	9.5

Source: own calculations based on survey data. hh = household(s).

**Table 2:** Correlation between insect and animal (meat, fish, milk, eggs) protein consumption, income, and insect relative to animal protein consumption, per capita.

Pearson Correlation Coefficient			
	Cash Income	Insect Protein	Relative Insect Protein
Insect protein	−0.061		
Relative insect protein	−0.177 **	0.634 **	
Animal protein	0.546 **	0.098	−0.288 **

Source: own calculations based on survey data; ** significant at 0.01 level.

**Table 3:** Correlation between insect protein consumption per capita, household size, farm size, and hours harvested per household.

Pearson Correlation Coefficient			
	Household Size	Farm Size	Insect Protein
Insect protein	−0.073	0.144 *	
Hours harvested	0.316 **	0.190 **	0.369 **

Source: own calculations based on survey data; * significant at 0.05, ** significant at 0.01 level.

**Table 4:** Multiple linear regression model summary; method: standard; one influential case excluded (*n* = 215), dependent variable: log of insect consumption per capita in kg.

R	R Square	Adjusted R Square	Std. Error of the Estimate	*F*	Sig.	Durbin-Watson
0.740	0.548	0.535	1.310	41.968	0.000	1.910

**Table 5:** Model coefficients.

Parameter	Coefficient B	Std. Error	T	Sig.	Part. Eta Squared
Constant	−3.527	1.330	−2.653	0.009	0.033
Log_HH size	−0.185	0.288	−0.642	0.522	0.002
Log_Animal protein	0.421	0.141	2.979	0.003	0.041
Log_Income capita	0.064	0.093	0.687	0.493	0.002
Log_Farm size	−0.020	0.106	−0.188	0.851	0.000
Rural/urban	0.298	0.576	0.517	0.606	0.001
Log_Harvest. time	0.480	0.070	6.822	0.000	0.183

Dependent variable: Log_Insectprotein.

**Figure A1:**
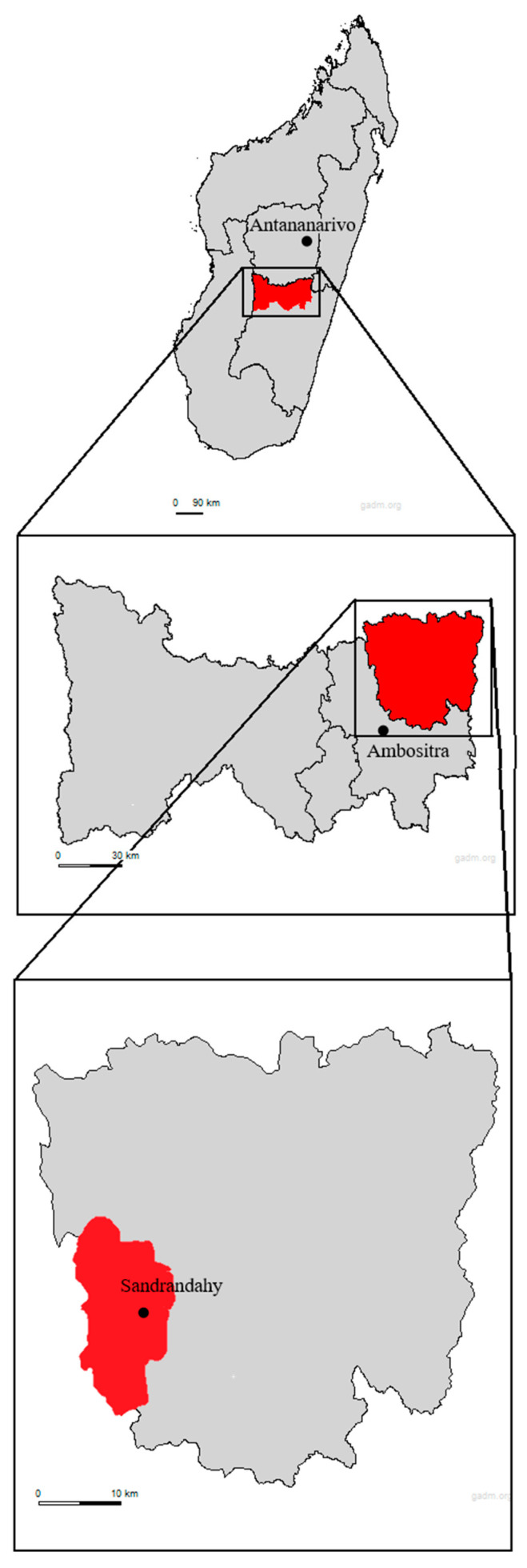
Location of Sandrandahy Commune within the district of Fandriana, the district within the region Amoron’i Mania, and the region within Madagascar. Source: ProciNut project by Sebastian Forneck; based on maps from Global Administrative Areas (2018). GADM database of Global Administrative Areas, version 3.6. [online] URL: www.gadm.org (accessed on 20 May 2020).

## Data Availability

Data can be made available upon request.

## Appendix A

**Table A1:** Protein content of different insects found in the study area.

				DRY MATTER BASIS	FRESH MATTER BASIS		DATA USED	
Malagasy Name	English Name	Family	Order	*Protein (g/100 g EP)*	*Protein (g/100 g EP)*	*Moisture Content (%)*	*Protein (g/100 g EP)*	*References/Observations*
*Voanosy* (Adult)	Weevils	CURCULIONIDAE	COLEOPTERA				21.2	Data used from *Voangory*
*Voangory* (Adult)	Scarab beetles	SCARABAEIDAE	COLEOPTERA		28.9 13.4	74.1	21.2	Mean of Köhler et al. [16] and Yhoung-Aree & Viwatpanich [48]
*Sakivy* (Larvae)	Scarab beetles Weevils	SCARABAEIDAE CURCULIONIDAE	COLEOPTERA	49.2			14.8	Gosh et al. [15]: mean of two species; moisture content assumed: 70%
*Abado* (Pupae)	Scarab beetles Weevils	SCARABAEIDAE CURCULIONIDAE	COLEOPTERA				14.8	Data used from *Sakivy*
*Tsikovoka* (Adult)	Water beetles	DYTISCIDAE	COLEOPTERA		25.1 21.0	49.1 61.2	23.0	Mean of Shantibala et al. [49] and Yhoung-Aree et al. [28]
*Jorery* (Adult)	Cicadas	CICADIDAE	HEMIPTERA	47.2			14.2	Raksakantong et al. [50]; moisture content assumed: 70%
*Valala* (Adult)	Grasshoppers Locusts	ACRIDIDAE OEDIPODINAE	ORTHOPTERA		14.3 20.1	76.7 69.0	17.2	Mean of Yhoung-Aree & Viwatpanich [48] and Oonincx & van der Poel [51]
*Akitra* (Adult)	Crickets	GRYLLIDAE	ORTHOPTERA		14.7	70.8	14.7	Yhoung-Aree & Viwatpanich [48]; mean for different crickets
*Zana-dandy* (Larvae)	Domesticated silkworms	BOMBYCIDAE	LEPIDOPTERA		18.9 26.6 12.2	69.9 75.3	19.2	Mean of Frye & Calvert [52] and Köhler et al. [16] and Yhoung-Aree & Viwatpanich [48]
*Landibe* (Larvae)	Wild silkworms	LASIOCAMPIDAE	LEPIDOPTERA		14.7	70.8	19.2	Data used from *Zana-dandy*

Sources: Taxonomy by Andrianantenaina Razafindrakotomamonjy (ProciNut project); own calculations based on given references; EP: edible portion.

**Table A2:** Protein content of different animal-based and plant-based foods. Composition (pour 100 g de partie comestible/100 g edible parts).

Aliments/Foods	Protein (g)
*Animal proteins*	
Viande de boeuf/Beef	14.60
Viande de porc/Pork meat	18.70
Viande de poulet/Poultry meat	12.30
Poissons frais/Fresh fish	10.30
Poissons seches/Dried fish	22.90
Lait frais/Fresh milk	3.10
Oeuf/Eggs	10.30
*Plant proteins*	
Tsaramaso mainty (sp vulgaris)/Dried beans	14.13
Pois du cap/Peas	24.05
Voanjobory/Bambara nuts	17.21
Kabaro fotsy (sp lunatus)/Lima beans	34.88
Kabaro sadamena (sp lunatus)	25.90
Kabaro mena (sp lunatus)	17.45
Kabaro mainty (sp lunatus)	18.93
Kabaro maramainty (sp lunatus)	20.81
Kabaro/Lima beans (average)	23.59
Arachide nature/Peanuts	29.91
*Rice*	
Paddy	6.00

Source: Organisation Nationale de Nutrition (ONN, Madagascar), without date.
